# Association of anterior and posterior occlusal planes with different Angle and skeletal classes in permanent dentitions: A lateral cephalometric radiograph study

**DOI:** 10.1007/s00056-018-0139-z

**Published:** 2018-05-17

**Authors:** Aleš Čelar, Ekrem Tafaj, Alexandra Graf, Stefan Lettner

**Affiliations:** 1 University Clinic of Dentistry, Orthodontics, Medical University of Vienna, Sensengasse 2a, 1090 Vienna, Austria; 2 Center for Medical Statistics, Informatics and Intelligent Systems, Medical University of Vienna, Spitalgasse 23, 1090 Vienna, Austria; 3 Austrian Cluster for Hard Tissue and Biomaterial Research, Karl Donath Laboratory, Medical University of Vienna, Sensengasse 2a, 1090 Vienna, Austria

**Keywords:** Anterior occlusal plane, Posterior occlusal plane, Malocclusion, Skeletal pattern, Angle classification, Anteriore Okklusionsebene, Posteriore Okklusionsebene, Malokklusion, Skelettales Muster, Angle-Klassifikation

## Abstract

**Objectives:**

Malocclusions affect about two-thirds of the population and orthodontic treatment is justified in 65% of these. However, the associations between anterior and posterior occlusal plane (AOP, POP) inclinations and Angle classification are lacking.

**Patients and methods:**

In a retrospective study, lateral cephalometric radiograph tracings of 230 previously untreated Caucasians, aged 13 to 49 years, yielded inclines of the bisector occlusal plane, AOP, and POP. All inclinations were referenced to the Sella-Nasion line and the Frankfort horizontal and were assigned to the Angle classification as well as skeletal groups (retrognathic, neutral, prognathic). Quantile regressions were calculated.

**Results:**

In the skeletal groups the angles between Sella-Nasion line and both AOP and POP were significantly different between the groups (*p* < 0.01), showing steep inclines in skeletal class II and flat inclines in skeletal class III. The angles Frankfort horizontal-to-POP and Frankfort horizontal-to-AOP showed the same trends but only the latter differed significantly between the groups (*p* = 0.02). Among the Angle groups, AOP inclinations did not differ significantly for both reference planes whereas POP inclinations were significantly different (*p* = 0.01 to Frankfort horizontal, *p* = 0.02 to Sella-Nasion). Angle class I patients showed the flattest POP.

**Conclusion:**

Occlusal plane inclines, measured to Sella-Nasion, were more consistent than those referenced to Frankfort horizontal. Sella-Nasion related anterior and posterior occlusal plane inclinations were steep in skeletal class II and flat in skeletal class III patients over all quantiles. Using the Angle classification, anterior and posterior occlusal plane inclinations did not follow this principle.

## Zusammenfassung

**Ziele:**

Etwa zwei Drittel der Bevölkerung sind von Malokklusionen betroffen, wobei eine kieferorthopädische Therapie bei 65% der Patienten indiziert ist. Bislang stehen jedoch keine Daten für die Beziehung zwischen anteriorer bzw. posteriorer Okklusionsebene (AOP bzw. POP) und Angle-Klassifikation zur Verfügung.

**Patienten und Methoden:**

In einer retrospektiven Studie lieferten die Durchzeichnungen lateraler Fernröntgenseitbilder von 230 unbehandelten Europäern (Alter 13 bis 49 Jahre) Neigungswinkel der gemittelten Okklusionsebene, der AOP und POP. Als Referenzebene dienten die Sella-Nasion-Linie und die Frankfurter Horizontale. Die Neigungen der Okklusionsebenen wurden sowohl zur bestehenden Angle-Klasse als auch zum skelettalen Muster (retrognath, neutral, prognath) in Beziehung gesetzt und mittels Quantilsregressionen analysiert.

**Ergebnisse:**

In den skelettalen Klassen bestanden signifikante Gruppenunterschiede für die Winkel zwischen Sella-Nasion-Linie und AOP als auch POP (*p*< 0.01). Steile Okklusionsebenenneigungen kennzeichneten die skelettale Klasse II, flache die skelettale Klasse III. Zur Frankfurter Horizontalen zeigten AOP und POP dieselben Trends, jedoch bestanden signifikante Gruppenunterschiede nur für die Neigung der AOP zur Frankfurter Horizontalen (*p* < 0.01). Bei Einteilung nach der Angle-Klassifizierung fanden sich für AOP und POP unabhängig von der Referenzebene keine signifikanten Gruppenunterschiede.

**Schlussfolgerung:**

Die Untersuchung bestätigte die Assoziation zwischen skelettalem Muster und der Neigung der POP über drei Quantile und zeigte diese Assoziation auch für die AOP-Neigung. Sella-Nasion-Messungen verdeutlichten steilere AOP und POP bei Patienten mit skelettaler Klasse II und eine flache Ausrichtung bei skelettaler Klasse III. Bei Einteilung nach der Angle-Klassifizierung bestand dieses Neigungsprinzip jedoch nicht. Kephalometrische Messungen mit Bezug zur Frankfurter Horizontalen sollten mit Zurückhaltung eingesetzt werden, SN-bezogene Messungen sind dagegen als verlässlicher einzuschätzen.

## Introduction

Malocclusions affect approximately two thirds of the population and orthodontic treatment can be justified in up to 65% of malocclusions [4, 19, 29]. Their etiology involves genetics, congenital abnormalities and syndromes, medical diseases, orofacial dysfunctions, habits, trauma, caries, anomalies of tooth number, tooth size and shape, and jaw size [20]. In the lateral aspect, the inclination of the occlusal plane can also influence the extent of anterior–posterior malocclusions, namely by the magnitude of the curve of Spee [5, 27] and the rotation of the occlusal plane. Prognathic mandibles showed flat occlusal planes [16, 23], whereas retrognathic mandibles showed an accentuated curve of Spee [1, 7, 18, 25].

Straight lines classically depict the occlusal plane in cephalometric tracings. For a better characterization of the sagittal curve of occlusion [17], Fushima et al. [7] divided the maxillary occlusal plane at the cusp tip of the maxillary second premolar into anterior and posterior occlusal planes (AOP, POP). Their inclinations were referenced to the Frankfort horizontal (FH) and showed a significantly steep POP in girls with skeletal class II.

Regarding the FH, variation in identifying orbitale and porion caused more error than tracing the sella-nasion line (SN) [30]. This aspect has not been considered in any study on the inclinations of the bisector occlusal plane, AOP, and POP, nor regarding the question of image quality of lateral cephalometric x-rays when measuring these inclinations. Furthermore, previous studies related AOP and POP to samples showing mandibular retrognathia and prognathia [2, 7, 28] but did not analyze the association of AOP and POP with the Angle classification.

Therefore, the aims of the present study were (1) to evaluate AOP and POP on high quality digital lateral cephalometric x‑rays of a contemporary European sample, (2) to also reference the AOP and POP inclinations to SN, (3) to analyze the relationship of these inclinations with the skeletal pattern and the Angle class, and (4) to use quantile regressions for exploration of data beyond means.

## Materials and methods

Our retrospective investigation was approved by the ethical board of the university and included pretreatment digital lateral skull radiographs of 230 previously untreated individuals, selected randomly from the files of the orthodontic department of the dental school, Medical University of Vienna, Austria. Radiographs and dental casts had been made for orthodontic treatment planning. X‑rays had been taken between 2004 and 2014 on a Philips Bucky Diagnost VS (Philips Healthcare, Eindhoven, The Netherlands) using 66–70 kV and 25–40 mAs at a 4 m focus receptor distance. The dose–area product ranged from 3–5 cGycm^2^. We used Fujifilm FCR XG-1 (Fuji Photo Film Co Ltd, Tokyo, Japan) for digital image visualization.

Appropriate participants had to meet the following eligibility criteria: complete permanent dentition without consideration of third molars and no former orthodontic treatment. Exclusion criteria were patients with a combination of unilateral Angle class II with class III occlusion, cleft lip or palate, craniofacial anomalies, oligodontia, previous orthodontics or orthognathic surgery, and prosthetic restorations with fixed partial dentures or implants.

The sample’s age ranged from 13–49 years (average 20.9 ± 8.7 years), comprising 95 males (mean age 20.1 ± 8.3 years, median 16.5 years) and 135 females (mean 21.5 ± 8.9 years, median 18.4 years). Forty-one individuals were Angle class I (18 male, 23 female), 107 Angle class II (45 male, 62 female), and 82 Angle class III (32 male, 50 female). The Angle class was determined on plaster casts oriented in maximum intercuspation without reconstruction to a former time point as fully dentate arches should have prevented mesial molar movement. This approach justified the comparison of the present Angle class with the present AOP and POP inclinations. In individuals with unilateral class I, we considered the malocclusion of the other side for group assignment.

**Fig. 1:**
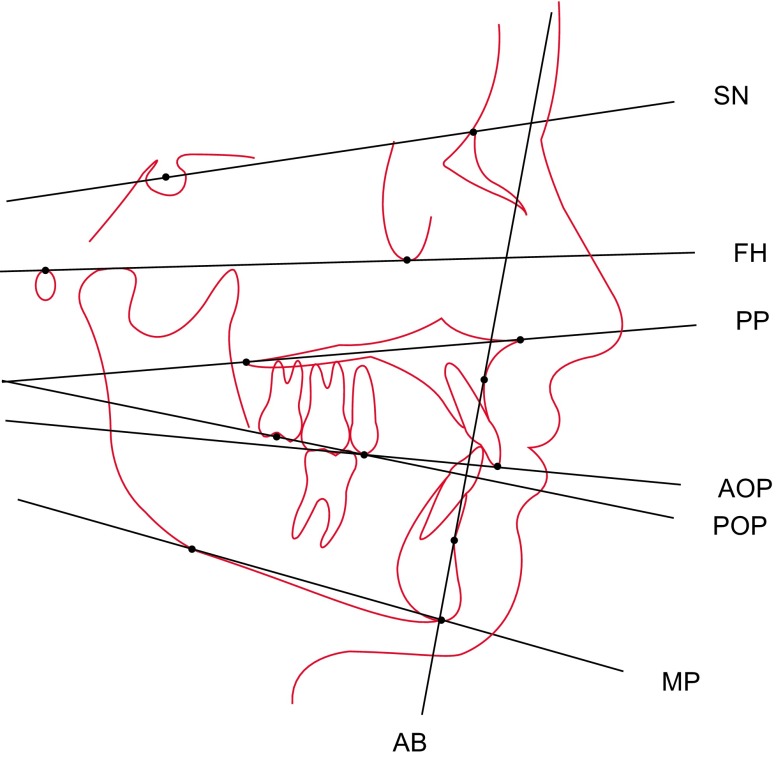
Illustration of the cephalometric tracings. *SN* sella-nasion*, FH *Frankfort horizontal*, PP *palatal plane*, AOP *anterior occlusal plane*, POP *posterior occlusal plane*, MP *mandibular plane*, AB *line between points A and B

Two operators made manual cephalometric tracings of the skull radiographs on acetate sheets with a drop-action pencil (lead width 0.5 mm). The light table (Just Normlicht, article no. 28860, Weilheim/Teck, Germany) was located in a silent, dimmed investigation room. As shown in Fig. 1, the operators identified SN, FH, the palatal plane (PP) from anterior nasal spine to posterior nasal spine, the mandibular plane (MP) from tangential gonion to menton, the nasion-pogonion line (NPg), and the line between points A and B (AB). The bisector occlusal plane (OP) was constructed by averaging the incisor and permanent first molar overbites [3]. AOP connected the maxillary incisal edge and the averaged cusp tip of the maxillary second premolar. POP linked the averaged cusp tip of the maxillary second premolar and the midpoint between the averaged cusp tips of the maxillary second molar [6, 28].

Further measurements encompassed Wits appraisal [9], overbite depth indicator (ODI) [11], angles FH-OP, FH-AOP, FH-POP, SN-AOP, SN-POP, and anteroposterior dysplasia indicator (APDI) [12]. For accurate determination, we measured the APDI as the posteriorly downward oriented angle between PP and AB instead of using the sum of three angles originally described by Kim [12].

The APDI value designated the sagittal maxillomandibular skeletal pattern for assignment to a neutral group (APDI between 77.5 and 85, *n* = 109), mandibular retrognathia (APDI < 77.5 *n* = 64), and prognathia (APDI > 85, *n* = 57) [12, 26]. Overall Kendall’s τ equalled 0.50 for the association between Angle classification and skeletal pattern.

Because of the retrospective character of the study, we assessed the reliability of the cephalometric measurements by comparison of our data with the original entries in the treatment planning data sheets. Discrepancies varied within 2.5° and 2 mm. In case of discrepancies above 1° or 1 mm, the operator measured the parameter in question again and selected the appropriate value. The intrarater reliability was assessed on 25 randomly selected x‑rays, repeatedly measured after 12 days. The intraclass correlation coefficients (ICCs) of SN-AOP, SN-POP, FH-AOP, FH-POP, APDI, and ODI ranged from 0.85–0.99. The interrater reliability was computed on the base of 37 radiographs and yielded ICCs between 0.86 and 0.97.

The data were then transferred to Microsoft Excel 2011 version 14.6 and checked for errors. Statistical computing and graphics were carried out in *R* (Version 3.2.4, R Core Team 2015a, R Foundation for Statistical Computing, Vienna, Austria), i.e., for descriptive statistics, quantile regressions and linear quantile mixed models with the operator as random effect to account for measurements from two observers [8, 14]. The results of the mixed model were similar to those of the model without random factor. Quantile regressions for the quantiles 0.25 (Q1), 0.5 (Q2) and 0.75 (Q3) were estimated for each of the dependent variables FH-OP, FH-AOP and FH-POP, including age, gender, and ODI as potential confounders and either skeletal class, Angle class, or APDI as covariates. Analogs of R^2^ were computed as proposed by Koenker and Machado [15]. Considering that FH values could be prone to measurement error, we recalculated our regressions for SN values as dependent variables and compared the results with respect to effect sizes and signs. Confidence intervals were computed by inverting rank tests. In the statistical models *p*-values are based on Wald tests as described by Koenker and Bassett [13] and were calculated with respect to the above-mentioned confounders. *P*-values given in Tables 1 and 2 are not conditioned on confounders and based on univariable models. ICCs are given according to Shrout and Fleiss [26].

**Table 1:** Descriptive statistics of the three skeletal groups: neutral (N, *n* = 109), mandibular retrognathia (Re, *n* = 64), and mandibular prognathia (Pro, *n* = 57)

	Skeletal group	Mean	SD	Median	IQR	Min	Max	Q1	Q3	*P*
Age	N	21.8	9.7	17.5	12	13	49.5	14	26	0.14
	Re	19.4	8	15.3	9.4	13	49.2	14	23.4	–
	Pro	20.7	6.9	18.4	10.7	13	41	15.3	26	–
FH-OP	N	7.5	4.9	8	5.5	− 4	22.7	5	10.5	0.03
	Re	8.6	4.7	8.8	5	0	22.5	6.2	11.3	–
	Pro	6	4.6	6	6	−4.5	19	3	9	–
FH-AOP	N	9.1	5.1	9	6.5	−1.8	21	5	11.5	<0.01
	Re	11	5.3	11	8	0	22	7	15	–
	Pro	6.6	4.4	6.5	5	−6	16	4	9	–
FH-POP	N	14.6	5.5	14	7.5	1	27	11	18.5	0.14
	Re	16	6.6	16	8.5	2	31.3	12.1	20.6	–
	Pro	14.2	5.6	13	6.5	2	27	11.5	18	–
SN-AOP	N	17.5	6	18	7	1	31	14.5	21.5	<0.01
	Re	19.8	5	20	5.6	6	30	17	22.6	–
	Pro	15.1	4.8	15	7.2	4	24.5	11.5	18.7	–
SN-POP	N	23.1	5	23	6.5	11	37.5	20	26.5	<0.01
	Re	25.1	5.6	25.2	7.6	13	38	22	29.6	–
	Pro	22.4	4.7	22	6.8	10.5	35	19.2	26	–
ODI	N	73.4	7.1	74	10	55	91.8	68.3	78.3	<0.01
	Re	74.6	9	73	11.2	47	94.7	68.9	80.1	–
	Pro	64.6	8.1	63	12	49.5	80.6	58	70	–
APDI	N	80.9	2.1	81	3.6	77.5	85	79	82.6	<0.01
	Re	73.1	3.3	74	4.4	62	77	71.2	75.6	–
	Pro	91.4	3.4	91	4	86	101	89	93	–
Wits	N	0.5	3	0	3.5	−6	9	−1.5	2	<0.01
	Re	3.6	3.2	3	4.2	−5	10	1	5.2	–
	Pro	−5.5	4.1	−5	6	−16	1	−8	−2	–

*SD* standard deviations, *IQR* interquartile ranges, *Min* minima, *Max* maxima, *Q1, Q3 *0.25 and 0.75 quantiles

*P*-values refer to difference of medians between the skeletal groups according to Wald tests

**Table 2:** Descriptive statistics for the groups Angle class I (*n* = 41), class II (*n* = 107), class III (*n* = 82)

	Angle class	Mean	SD	Median	IQR	Min	Max	Q1	Q3	*P*
Age	I	20.9	9.2	16.5	11.7	13	49.1	14	25.7	0.92
	II	21.3	9.4	16.9	12.5	13	49.5	14	26.5	–
	III	20.3	7.3	17.5	9.4	13	46.9	14.3	23.8	–
FH-OP	I	8	4.1	7	6	1	17	5	11	0.62
	II	7	4.9	7.8	6.2	−4.5	21	3.8	10	–
	III	7.8	5.2	7	5.4	−4.5	22.7	5	10.4	–
FH-AOP	I	10.3	5.3	9	7	1.5	21	7	14	0.35
	II	9.1	5.5	9.5	8.2	−2.5	22	4.8	13	–
	III	8.3	4.8	8	6.2	−6	21	4.8	11	–
FH-POP	I	13.6	6.1	12.5	8	1	27	10	18	0.24
	II	14.9	5.7	14	6.1	2	31.3	12	18.1	–
	III	15.5	6	14.8	7.9	2	27	12	19.9	–
SN-AOP	I	18.3	6.1	19	7	1.3	30	15	22	0.35
	II	17.7	5.9	18	7.1	1	31	14	21.1	–
	III	17.1	5.1	17	7.9	4	28	13.1	21	–
SN-POP	I	22.4	5.5	22.3	10	13	31	18	28	0.54
	II	23.6	5.3	23.2	7.2	11	38	19.8	27	–
	III	24	5	24	5.9	10.5	37.5	21	26.9	–
ODI	I	73.6	7	74	7	58.9	91.8	70	77	<0.01
	II	74.7	8.2	75	10.2	47	94.7	69.8	80	–
	III	66.3	8.1	66.5	11.6	49.5	84	60.1	71.8	–
APDI	I	80.7	5.1	80.5	4	66	91.5	78	82	<0.01
	II	77.4	5.6	77.8	7	62	91	74	81	–
	III	86.7	6.7	86.6	10.7	69	101	81.7	92.4	–
Wits	I	0.2	2.8	0	3.5	−6.5	8	−1.5	2	<0.01
	II	2.5	3.6	2.5	4.5	−7	10	0.5	5	–
	III	−3.7	4.5	−2.8	5.5	−16	5	−6	−0.5	–

*SD* standard deviation, *IQR* interquartile ranges, *Min* minima, *Max* maxima, *Q1, Q3 *0.25 and 0.75 quantiles

*P*-values refer to difference of medians between the Angle groups according to Wald tests

## Results

The average POP was approximately 6° steeper than the average AOP. Means and medians of the measurements FH-OP, FH-AOP, FH-POP, SN-AOP, and SN-POP differed 0–2.3° between the Angle groups and 0.4–5° between the skeletal groups. Between-group differences of Q1 and Q3 ranged from 0–3° (Angle classification) and 0.5–6° (skeletal type). Tables 1 and 2 show the descriptive statistics separately for the Angle and the skeletal classifications. Table 3 lists the coefficients of regression and confidence intervals, and Table 4 the analogs of the coefficient of determination. The ICCs for repeated measurements yielded 0.99 for SN-AOP, 0.95 for FH-AOP, 0.96 for SN-POP, and 0.86 for FH-POP.

**Table 3:** Regression coefficients and 95% confidence intervals (limits in parentheses) for the FH-POP, SN-POP, FH-AOP, and SN-AOP statistical models at the 0.25, 0.5, and 0.75 quantiles (Q1, Q2, Q3)

	FHPOP			SNPOP			FHAOP			SNAOP		
Covariates	Q1	Q2	Q3	Q1	Q2	Q3	Q1	Q2	Q3	Q1	Q2	Q3
ODI, retrognathia, and prognathia												
– Intercept	**12.07 (4.36, 18.19**)	**17.61 (4.64, 23.14)**	**28.37 (10.92, 32.62)**	**33.06 (25.7, 37.73)**	**27.91 (22.59, 35.32)**	**38.09 (26.77, 47.88)**	−0.7 (−7.91, 2.43)	−0.41 (−6.25, 4.53)	2.46 (−5.85, 12.35)	**18.96 (7.06, 22.52)**	**18.48 (14.24, 26.65)**	**24.04 (17.56, 31.33)**
– Age (10 year increment)	**0.51 (0.08, 1.71)**	0.29 (−0.97, 1.03**)**	−0.39 (−0.88, 1.15)	0.41 (−0.6, 1.04)	0.17 (−0.63, 0.98)	−0.53 (−1.21, 0.73)	−0.48 (−1.48, 0.13)	−0.3 (−1.15, 0.7)	−0.16 (−0.88, 1.01)	**−1.98 (−2.62, −0.54)**	−0.62 (−1.72, 0.47)	0 (−0.83, 1.24)
– Gender (m)	−1.11 (−2.98, 0.83)	−0.03 (−1.47, 1.8)	1.04 (−0.98, 2.29)	−2.72 (−3.67, −1.44)	−0.97 (−2.11, 0.93)	−0.4 (−2.45, 1.64)	0.48 (−0.53, 1.79)	0.22 (−0.76, 1.53)	0.12 (−1.53, 1.45)	−0.63 (−1.71, 1.02)	−0.55 (−1.71, 0.92)	−0.87 (−1.95, 2.02)
– ODI	−0.02 (−0.11, 0.09)	−0.06 (−0.16, 0.15)	−0.13 (−0.17, 0.1)	−0.17 (−0.24, −0.07)	−0.07 (−0.18, 0.03)	**−0.13 (−0.27, −0.01)**	**0.09 (0.05, 0.19)**	**0.14 (0.06, 0.21)**	**0.13 (0.01, 0.25)**	−0.01 (−0.05, 0.14)	0.01 (−0.11, 0.06)	−0.03 (−0.13, 0.06)
– Retrognathic	**0.99 (0.67, 3.66)**	1.37 (−0.55, 4.2)	**3.14 (0.17, 2.84)**	**1.11 (0.16, 2.93)**	**2.91 (1.29, 4.92)**	**1.59 (0.31, 4.99)**	1.6 (−0.31, 4.04)	**1.92 (0.83, 3.7)**	2.21 (−0.06, 4.59)	**2.61 (0.18, 3.19)**	**2.37 (0.96, 3.89)**	1.31 (−0.04, 3.57)
– Prognathic	−0.26 (−1.55, 1.59)	−1.67 (−2.97, 2.07)	−0.57 (−1.67, 0.94)	**−1.88 (−3.72, −0.58)**	−0.84 (−2.97, 0.05)	−2.73 (−5.07, 0.34)	−0.12 (−2.01, 1.51)	**−1.59 (−3.29, −0.05)**	−1.81 (−4.08, 0.23)	**−2.75 (−3.97, −0.6)**	**−2.34 (−5.32, −1.12)**	−3.6 (−5.58, 0.09)
ODI, Angle class II, and Angle class III												
– Intercept	**9.69 (1.44, 14.77)**	**10.01 (3.64, 18.18)**	**10.84 (3.57, 27.98)**	**26.47 (13.26, 31.06)**	**21.04 (17.16, 34.6)**	**32.46 (25.69, 40.67)**	−2.79 (−9.1, 2.76)	−5.23 (−10.73, 2.17)	−3.52 (−8.54, 1.43)	**11.31 (1.86, 24.29)**	**16.84 (8.26, 19.84)**	**18.82 (16.72, 24.15)**
– Age (10 year increment)	0.1 (−0.51, 1.1)	0.49 (−1, 1.45)	−0.06 (−1, 1.24)	−0.04 (−0.83, 0.85)	−0.01 (−0.83, 0.63)	−0.37 (−1.25, 1.15)	**−0.9 (−1.81, −0.07)**	−0.8 (−2.11, 0)	0.04 (−0.74, 1.05)	**−1.61 (−2.53, −0.75)**	−0.68 (−2.02, 0.15)	−0.39 (−0.82, 0.31)
– Gender (m)	−0.06 (−2.92, 0.88)	−0.05 (−2.31, 1.31)	0.95 (−1.13, 2.94)	**−2.55 (−3.41, −0.94)**	**−1.34 (−2.51, −0.16)**	−0.09 (−2.11, 1.12)	0.2 (−0.62, 1.2)	−0.01 (−1.58, 1.68)	−0.04 (−1.16, 1.68)	−0.31 (−2.83, 0.58)	0.3 (−1.96, 1.4)	−0.53 (−1.5, 1.51)
– ODI	0 (−0.05, 0.13)	0.02 (−0.09, 0.1)	0.1 (−0.12, 0.15)	−0.09 (−0.14, 0.09)	0.02 (−0.12, 0.06)	−0.05 (−0.17, 0.03)	**0.15 (0.1, 0.22)**	**0.21 (0.11, 0.29)**	**0.23 (0.16, 0.28)**	0.08 (−0.05, 0.21)	0.04 (−0.01, 0.18)	**0.05 (0.01, 0.11)**
– Angle class II	**2.07 (0.37, 3.48)**	1.4 (−0.33, 2.97)	0.06 (−2.75, 3.85)	1.49 (−0.16, 3.12)	1.52 (−0.6, 2.5)	−1.29 (−2.35, 2.35)	**−1.64 (−2.37, −0.09)**	0.1 (−2.57, 2.69)	−0.59 (−5.77, 1.22)	0.42 (−4.13, 1.68)	−0.77 (−3.11, 1.19)	−0.85 (−2.51, 0.52)
– Angle class III	1.92 (−0.34, 3.87)	**2.73 (0.86, 4.4)**	3.18 (−0.67, 6.55)	1.26 (−0.59, 3.93)	1.86 (−1.61, 3.41)	−2.02 (−3.13, 2.28)	−0.19 (−1.24, 1.38)	1.11 (−1.54, 3.33)	−0.5 (−5.57, 1.1)	0.27 (−4.55, 1.97)	−1.53 (−3.63, 2.23)	−0.34 (−2.83, 0.78)
ODI and APDI												
– Intercept	**25.02 (9.23, 30.44)**	**34.05 (10.34, 44.37)**	**34.92 (15.1, 66.73)**	**46.92 (36.4, 57.62)**	**52.27 (38.92, 60.23)**	**54.51 (38.37, 72.58)**	8.66 (−9.01, 16.43)	**15.97 (13.8, 25.13)**	12.95 (−0.59, 41.45)	**37.76 (25.01, 50.2)**	**39.28 (27.58, 52.06)**	**34.49 (24.91, 57.58)**
– Age (10 year increment)	**0.56 (0.26, 1.43)**	0.4 (−1.95, 1.35)	0.73 (−1.19, 0.97)	0.37 (−0.25, 0.87)	−0.19 (−0.59, 0.56)	−0.52 (−1.25, 0.61)	−0.6 (−1.58, 0.02)	−0.54 (−1.07, 0.04)	−0.37 (−0.84, 0.88)	**−1.79 (−2.5, −0.82)**	−0.72 (−1.83, 0.28)	−0.2 (−0.67, 0.9)
– Gender (m)	−1.36 (−2.87, 0.52)	−0.31 (−1.3, 1.91)	0.32 (−0.92, 3.19)	**−2.45 (−3.66, −1.48)**	−0.83 (−2.37, 0.68)	−0.18 (−2.98, 1.98)	0.62 (−0.82, 1.36)	−0.08 (−0.67, 1.1)	−0.15 (−1.55, 1.81)	−0.62 (−1.81, 0.45)	−0.11 (−2.1, 0.85)	−0.29 (−1.28, 1.92)
– ODI	−0.04 (−0.09, 0.04)	−0.08 (−0.13, 0.06)	−0.06 (−0.23, 0.07)	**−0.19 (−0.25, −0.11)**	**−0.12 (−0.16, −0.07)**	**−0.12 (−0.23, −0.02)**	**0.07 (0.04, 0.18)**	**0.12 (0.04, 0.16)**	0.19 (−0.01, 0.26)	0 (−0.06, 0.12)	−0.03 (−0.09, 0.06)	0.02 (−0.14, 0.07)
– APDI	−0.15 (−0.19, −0.01)	−0.18 (−0.26, −0.05)	−0.16 (−0.3, 0.02)	**−0.16 (−0.29, −0.08)**	**−0.25 (−0.29, −0.13)**	**−0.22 (−0.38, −0.12)**	−0.09 (−0.16, 0.07)	**−0.17 (−0.23, −0.15)**	**−0.17 (−0.34, −0.05)**	**−0.24 (−0.33, −0.11)**	**−0.22 (−0.35, −0.16)**	**−0.17 (−0.4, −0.06)**

Values in bold do not include zero in the confidence interval and indicate statistical significance

“Intercept” refers to the expected mean of an imaginary patient whose values are zero (age 0 years, APDI 0, ODI 0)

*ODI* overbite depth indicator, *APDI* anteroposterior dysplasia indicator

**Table 4:** Analogs of R^2^ as approximation to the coefficients of determination of all statistical models for FH-POP, SN-POP, FH-AOP, and SN-AOP at the 0.25, 0.5 and 0.75 quantiles Q1, Q2, Q3

	Q1	Q2	Q3
FH-AOP (APDI)	0.06	0.09	0.1
SN-AOP (APDI)	0.08	0.06	0.04
FH-POP (APDI)	0.03	0.01	0.01
SN-POP (APDI)	0.07	0.05	0.05
FH-AOP (Angle class)	0.06	0.05	0.08
SN-AOP (Angle class)	0.05	0.02	0.02
FH-POP (Angle class)	0.02	0.01	0.02
SN-POP (Angle class)	0.06	0.02	0.01
FH-AOP (skeletal group)	0.06	0.1	0.1
SN-AOP (skeletal group)	0.09	0.07	0.04
FH-POP (skeletal group)	0.02	0.01	0.02
SN-POP (skeletal group)	0.07	0.05	0.05

Parameter of interest in parentheses (APDI value, Angle class, skeletal maxillomandibular relationship)

In the Angle groups the FH-AOP and SN-AOP means were greatest in class I patients and lowest in the class III group. The highest FH-POP and SN-POP means and medians were found in the class III group, the lowest FH-POP and SN-POP means and medians in the Angle class I group. Between-group differences of the FH-OP, FH-AOP, FH-POP, SN-AOP, and SN-POP medians were not significantly different within the Angle classification.

Assigned to the skeletal pattern, the group with mandibular retrognathia showed the highest means, medians, Q1 and Q3 values for FH-OP, FH-AOP, FH-POP, SN-AOP, and SN-POP, whereas the prognathic group displayed the lowest ones with one exception (Q1 for FH-POP was lowest in the neutral group). The medians’ between-group differences of OP, AOP, and POP were significantly different (*p* < 0.05) except FH-POP.

Regarding the average vertical jaw relationship, the ODI specified that the Angle class III group and the prognathic group were hyperdivergent. The neutral and retrognathic groups as well as the Angle class I and class II groups were normodivergent. The Wits appraisal values conformed to the skeletal group assignment by APDI (Pearson’s r = −0.78).

### Bisector occlusal plane quantile regressions

In order to broaden the statistical evaluation beyond means and medians, we added the τ = 0.25 (Q1) and τ = 0.75 quantiles (Q3) for the presentation of our regressions. Age had a slight but significant negative effect on FH-OP only in the lower quantiles (low FH-OP values decreased with age) but no significant effect on FH-OP in the middle and high spectrum of the distribution. Gender played practically no role. FH-OP slightly increased with ODI without significance.

There was a statistically significant effect of the APDI on FH-OP at the median (*p* = 0.01). Comparing τ = 0.25, 0.5, 0.75, the relation between APDI and FH-OP was not significantly different over the distribution. Mandibular prognathism lowered and mandibular retrognathism increased FH-OP consistently for all quantiles.

Inclusion of the Angle class instead of the skeletal pattern did not yield significant effects of age, gender, and ODI. There was no significant influence from the Angle groups on FH-OP over the quantiles.

### Anterior occlusal plane quantile regressions

The effects of age and gender on FH-AOP were negligible. ODI had a strong positive association with FH-AOP values at all quantiles in the skeletal and Angle groups (*p* < 0.01) but a not significant association with SN-AOP. ODI did not significantly affect SN-AOP (*p* = 0.5) in the Angle groups.

The skeletal groups showed small effects for FH-AOP, though significant for the median (*p* < 0.01 at τ = 0.5) and similar over the quantiles (*p* = 0.39 comparing τ = 0.25, 0.5, 0.75). The retrognathic group yielded higher values for SN-AOP and the prognathic group lower values at all quantiles without different effects (*p* < 0.01 for τ = 0.5, *p* = 0.38 comparing τ = 0.25, 0.5, 0.75).

**Fig. 2:**
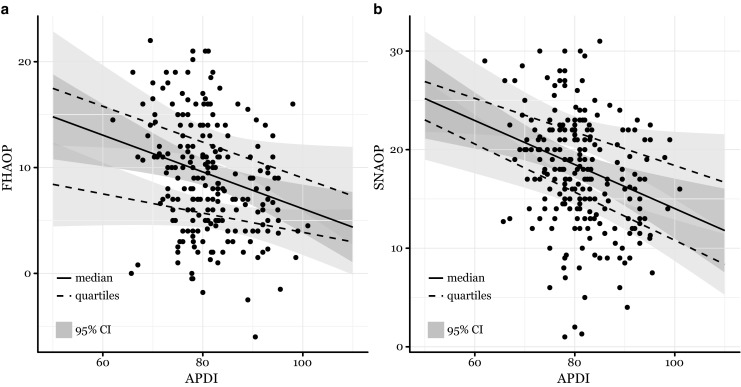
Graphic comparison of the linear estimates for the lower and upper quantiles (*dashed lines*), medians (*solid line*), and 95% confidences intervals (95% CI, *grey areas*). *FHAOP* Frankfort horizontal–anterior occlusal plane, *APDI* anteroposterior dysplasia indicator, *SNAOP *sella-nasion–anterior occlusal plane

The Angle groups did not affect FH-AOP or SN-AOP. The APDI showed effects on FH-AOP (*p* < 0.01 for τ = 0.5, *p* = 0.22 comparing τ = 0.25, 0.5, 0.75) and SN-AOP (*p* < 0.01 for τ = 0.5, *p* = 0.41 comparing τ = 0.25, 0.5, 0.75). Fig. 2 depicts the relations between APDI and FH-AOP as well as APDI and SN-AOP.

### Posterior occlusal plane quantile regressions

Age and gender did not significantly influence FH-POP or SN-POP. ODI had a small but not significant effect on SN-POP when accounting for the skeletal groups (*p* = 0.15), which did not show substantial effects on FH-POP as well (neither for τ = 0.5 nor τ = 0.25, 0.5, 0.75). The effects of the skeletal group on SN-POP were more pronounced and statistically significant for the median and homogenous over three quantiles. The mandibular prognathia group and the neutral group resulted in lower SN-POP values and the mandibular retrognathia group in higher ones (*p* < 0.01).

Using the Angle groups FH-POP remained almost the same at the lower quantiles. In the mid and higher quantiles, the Angle class II and Angle class III groups were associated with higher but not significant FH-POP values (*p* = 0.2 for τ = 0.5, *p* = 0.6 comparing τ = 0.25, 0.5, 0.75). SN-POP yielded similar results (*p* = 0.46). When tested for APDI, SN-POP resulted in a strong negative association (*p* < 0.01 for τ = 0.5) and significantly stronger effects for higher quantiles (*p* = 0.01 comparing τ = 0.25, 0.5, 0.75). Testing FH-POP and APDI, we observed a high negative association with increasing FH-POP values as well (*p* = 0.03 for τ = 0.5) but not significant differences of effects over the distribution (*p* = 0.96 comparing τ = 0.25, 0.5, 0.75).

## Discussion

The cant of the occlusal plane describes a vertical morphologic trait, which then may affect the anteroposterior jaw position. In the present study significant between-group differences of FH-AOP, SN-AOP, and SN-POP confirmed what Fushima et al. [7] and Tanaka and Sato [28] had found for the POP in their skeletal groups: steep POP in skeletal class II, flat POP in skeletal class III. While former studies had only used the Frankfurt horizontal as the plane of reference [7, 23, 28, 31], we added the sella-nasion line for a second set of data. The SN-referenced measurements not only endorsed the significant association between skeletal pattern and orientation of the bisector occlusal plane or the POP but also showed it for the AOP. At almost all quantiles SN-AOP and FH-AOP followed the same principles of inclination as POP if a skeletal classification was used.

Conversely the Angle groups did not respect the principles of occlusal plane steepness or flatness and did not show statistically significant between-group differences. Also in the regressions, the Angle classes did not significantly affect the occlusal plane inclinations.

The quantile computations demonstrated constant effects of the skeletal groups over the whole distribution of SN-AOP and SN-POP. Contrarily widespread unambiguous trends did not exist for the ODI. Despite some significant ODI effects on FH-AOP and SN-POP, steeper occlusal planes for higher ODI prevailed mostly in the higher quantiles, whereas lower APDIs were associated with steeper occlusal planes over all quantiles. Using ANOVA and post hoc tests, also Tanaka and Sato did not describe significant effects of the ODI on the cant of the occlusal plane in mature permanent dentitions [28]. Their and our results suggest a greater impact of the anteroposterior skeletal pattern on the inclinations of OP, AOP, and POP, although the vertical dimension has been thought to mainly influence the occlusal plane inclination [24].

Flat POPs of the Angle class I group resembled the POPs of the skeletal class III group in our study. This analogy could be ascribed to minor differences of the dentoalveolar structure between some Angle class I and some skeletal class III patients. Reyes et al. [21] compared normal occlusion in 5‑ to 16-year-old Caucasians with untreated class III Caucasians of the same age. The dentoalveolar differences between class I and class III subjects were sporadic and the main dentoalveolar finding was a larger extrusion of class III maxillary molars at ages 11–15 years. More maxillary molar eruption fits the flat POPs of our skeletal class III group but not the steep POPs of our Angle class III group.

Different outcomes between Angle classification and skeletal pattern indicate a certain independence of the molar relationship from the facial skeleton. The correlation between these two classifications was moderate in our study. Keeling et al. [10] matched the features of occlusion and craniofacial morphology and found a poor association in 9‑ to 16-year-olds, i.e*.*, specific skeletal types did not show typical occlusal categories or combinations. Furthermore, occlusion did not reflect the sagittal position of either the maxilla or mandible, and the highest correlations between molar relation and skeletal measurements explained 31% of the variance [10]. Regarding the extent of the curve of Spee, cephalometric measurements explained 34% of its total variance [5]. Consequently, other factors than craniofacial shape might influence the cant of the occlusal plane, for example myofunction, jaw function, extent of tooth eruption, and anterior or posterior dental crowding [21–23, 25].

Based upon geometric morphometrics of lateral cephalometric tracings, the cranial shapes of individuals with normal occlusion and malocclusion overlapped without formation of distinct clusters [6]. In other words, extensive variation of cranial shape did not substantiate a tight relation between craniofacial morphology and occlusal pattern. We consider biological variation and the aforementioned conditions to contribute to the differences of occlusal plane inclinations between Angle groups and skeletal groups.

In the quantile computations, the FH-related measurements showed considerably more uncertain behavior than the SN-referenced data. The confidence intervals of the SN measurements were often smaller than the FH referenced ones, and the ICCs of repeated measurements of SN-AOP and SN-POP higher than those of FH-AOP and FH-POP. Fig. 2 shows an increasingly negative relationship of SN-AOP with APDI over the quantiles, while FH-AOP shows the most extreme association in the middle quantile. This constellation could be explained by the potential weakness of FH-related measurements [30]. The identification of porion may have created more noise. In general measurements referenced to SN were more consistent and allowed easier interpretation. The exclusive use of FH-referenced data may also have precluded former studies from detecting an association between AOP and skeletal traits [7, 28].

From a clinical perspective, orthodontic treatment of the anteroposterior component of malocclusions could aim at changing the occlusal plane inclination, possibly facilitating the adaptation of the mandible toward a therapeutic position. The orientation of the occlusal plane should not dogmatically be considered as unchangeable because we found a range of occlusal plane angles up to 30°. However, esthetically undesirable occlusal plane angulations should be avoided [18] and the therapeutic change of the occlusal plane inclination necessitates further studies.

Limitations of the present investigation include the retrospective cross-sectional design, evaluation of the three-dimensional (3D) occlusal plane in two dimensions, and potential bias from averaging bilateral structures to single outlines. A two-dimensional lateral cephalometric radiograph will not fully explain the association between occlusal plane, facial skeletal traits, and Angle class. Asymmetric occlusal inclinations might alter the spatial position of the mandible because of transverse forces and resultant mandibular lateral deviation [2]. The 3D issues, the alteration of the occlusal plane angulation for channeling therapeutic goals, and consequences of such alterations on posttreatment stability should be addressed in further studies.

## Conclusions

Our study confirmed the association between skeletal pattern and the posterior occlusal plane inclination over three quantiles and also showed this association for the anterior occlusal plane inclination over the distribution: flat occlusion in untreated skeletal class III, steep in untreated skeletal class II. Within the Angle classification, there were no significant between-group differences and no unambiguous associations of steep or flat occlusal plane inclination, neither for the bisector occlusal plane nor the anterior or posterior occlusal planes. The effects of age and gender on the cant of the occlusal plane segments were small and almost negligible. Cephalometric measurements, which are related to the Frankfort horizontal, should be used with caution, whereas sella-nasion referenced measurements can be considered more reliable.
